# Lymphangitic Carcinomatosis of Possible Urothelial Origin

**DOI:** 10.7759/cureus.36030

**Published:** 2023-03-11

**Authors:** Tanjot Saini, Sundeep Bekal, Andrew D Liman

**Affiliations:** 1 Internal Medicine, University of California San Francisco Fresno, Fresno, USA; 2 Hematology and Oncology, Fresno VA Medical Center, Fresno, USA

**Keywords:** uro oncology, metastatic lesion, hypoxic respiratory failure, urologic cancer, covid 19

## Abstract

Urothelial cancer with lymphangitic carcinomatosis is a rare clinical phenomenon that is not commonly associated with acute respiratory failure. However, the recent prevalence of COVID-19 may predispose a patient’s respiratory system to become more susceptible to metastatic lymphangitic spread.

We present a case of a 57-year-old male with progressively worsening hypoxic respiratory failure after testing positive for COVID-19 six months prior. Imaging during the hospitalization showed adenopathy consistent with lymphangitic carcinomatosis that was not present six months prior. Acute respiratory deterioration is associated more commonly with infection rather than the progression of cancer, but infectious, autoimmune, and cardiac processes were deemed minimal contributory factors. The patient’s respiratory decline only allowed for a T-11 vertebral biopsy which showed poorly differentiated metastatic carcinoma of possible urothelial origin.

Urothelial cancer leading to respiratory failure due to lymphangitic carcinomatosis is an uncommon phenomenon, but in the setting of prior COVID-19, it may make the respiratory system more susceptible to lymphangitic spread. However, research is limited due to the recent prevalence of COVID-19, and more research is necessary to investigate a potential correlation with rapid lymphatic carcinomatosis after COVID-19 infection.

## Introduction

Bladder carcinoma is the fourth most common cancer in men and the ninth most common cancer in women in the United States. Ninety percent of bladder carcinomas are of urothelial origin, which originates from the urothelium and is typically composed of transitional cell carcinoma [1-2]. This epithelium starts from the renal pelvis, ureters, bladder, and urethra and can be treated in various methods depending on the staging by surgery or chemotherapy. One rare complication of urothelial cancer is pulmonary lymphangitic carcinoma (PLC), which is typically seen in adenocarcinoma of the primary lung, breast, and gastric cancers [3]. Pulmonary lymphangitic carcinomatosis typically presents with non-specific symptoms such as dyspnea and a dry cough leading to a delayed diagnosis [3].

PLC happens with the infiltration of cancer cells and inflammatory cells, leading to interstitial edema inside and surrounding lymphatic vessels [4]. This leads to obstruction of lymphatic drainage and causes retrograde migration of cancer cells into lung tissues or anterograde migration of cancer cells in the pleura into the pulmonary hilar lymph nodes [5]. A delayed diagnosis of a highly aggressive disease is a major component of why most patients die within two months of their respiratory symptoms [6].

Lymphangitic carcinomatosis represents poor prognosis in patients with malignancy and is often representative of end-stage severity. Though there are published reports about the occurrences of lymphangitic carcinomatosis, there is very little literature in the context of patients with unresolved COVID-19 symptoms as well as with an underlying urothelial malignancy.

## Case presentation

A 57-year-old-male with a medical history of hypertension, controlled diabetes type 2, and NSTEMI (non-ST-elevation myocardial infarction) with two stents initially presented to the emergency department with two days of pallor, emesis, and myalgias in September 2020. Laboratory values at the time included normal hemoglobin, white blood cell count, creatinine, liver enzymes, urinalysis, and a negative COVID PCR test, in addition to normal oxygen saturation. During this admission, he received a computed tomography (CT) abdomen and pelvis with IV contrast which was remarkable for nonspecific gastric wall thickening and colonic diverticulosis. Chest radiograph showed no acute pulmonary process, and the patient’s symptoms were determined to be secondary to viral gastroenteritis. Malignancy was not suspected at the time due to his acute onset of symptoms and lack of any lymphadenopathy or tumor burden identified on contrast-enhanced imaging. He received intravenous fluids and supportive care before discharge with close outpatient follow-up. A few months later, in December 2020, the patient contracted COVID-19 and tested positive on COVID polymerase chain reaction (PCR) testing but never required hospitalization. In late April 2021, the patient presented to the emergency department with chest pain and self-reported shortness of breath that never completely resolved back to baseline since his initial COVID-19 diagnosis a few months prior. A CT-pulmonary angiography (CT-PA) was completed and was negative for a pulmonary embolism but showed significant new mediastinal, hilar, and upper abdominal adenopathy with pulmonary nodules.

Shortly after, he was evaluated outpatient by our pulmonary specialists, who referred him back to the emergency department for acute hypoxic respiratory failure after he was found to have continued dyspnea and hypoxia. While in the emergency department, he endorsed continued shortness of breath and was found to be saturated in the mid-80s on room air but denied any urinary symptoms. Since his last admission two weeks prior, laboratory tests, including HIV PCR, coccidioidomycosis PCR, COVID-19 PCR, and a serum autoimmune panel, were all within normal limits.

**Figure 1:**
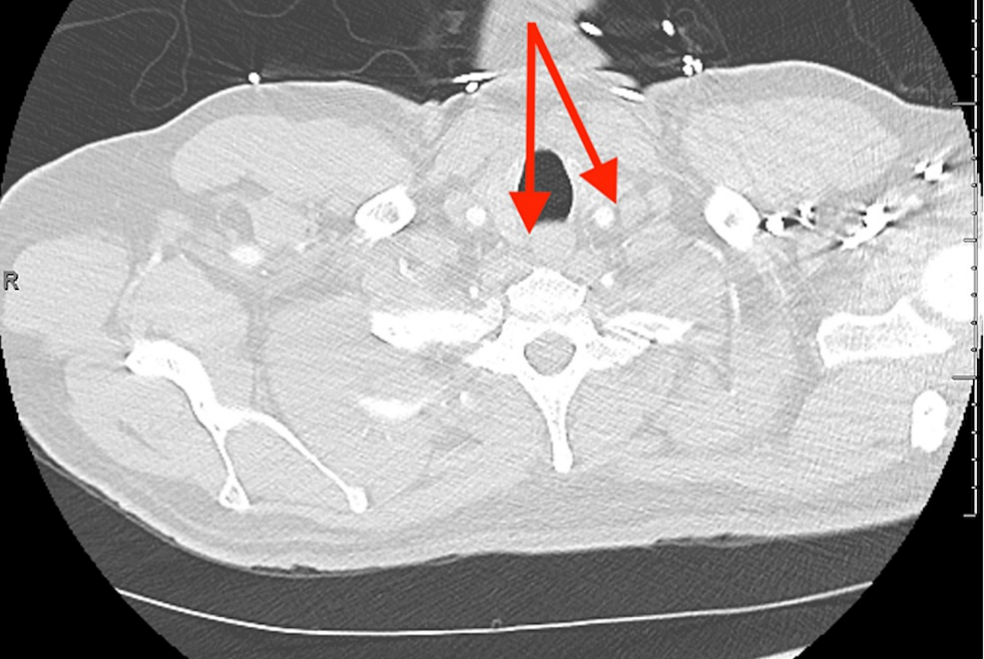
New lymphadenopathy on CT-PE

**Figure 2:**
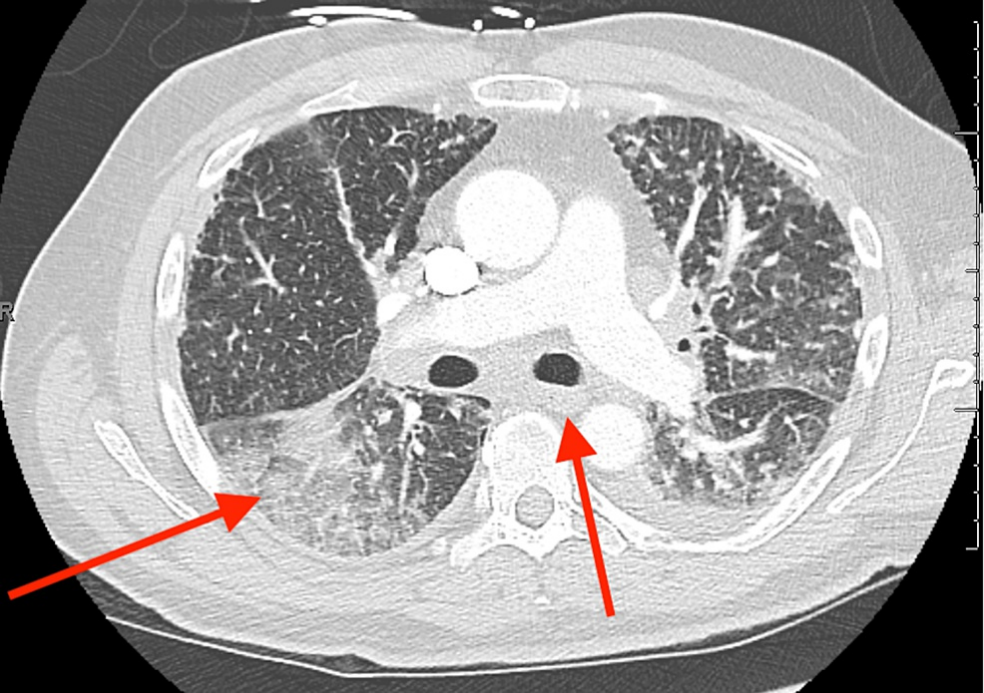
Ground glass opacities and new onset lymphadenopathy

While in the emergency department, he was evaluated by the inpatient cardiologists due to new precordial T wave inversions in an S1Q3T3 pattern (typical pattern for a pulmonary embolus on ECG) and an elevated troponin of 0.1 ng/mL (Ref. 0.00 - 0.04 ng/mL). Urinalysis was negative for any markers of infection, ketones, glucose, and bilirubin. Repeat CT-PA was negative for an embolus but showed diffuse parenchymal abnormality with diffuse interstitial prominence, nodular, and ground glass opacities throughout both lungs, remarkably increased since the CT-PA two weeks prior (Figures 1, 2).

**Figure 3:**
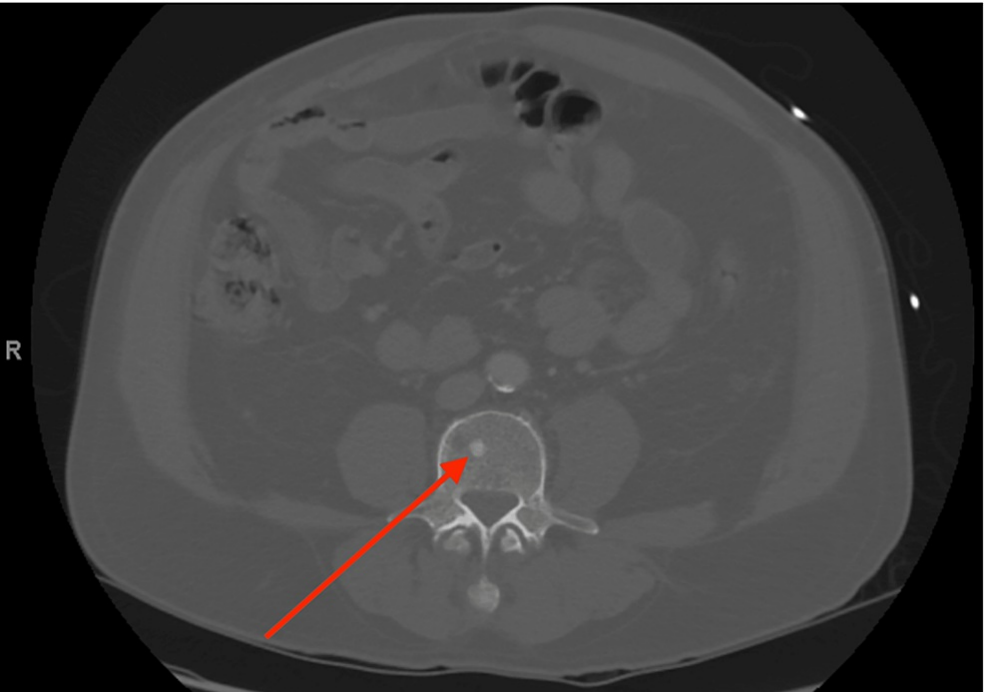
T11 biopsy site

A pharmacological stress test was completed to evaluate for ischemia of the left anterior descending artery but was ultimately unremarkable, with a left ventricular ejection fraction of 72% with normal wall motion. Initially, pulmonary edema was considered as a differential to the cause of the patient's acute respiratory failure. However, as the patient was nonedematous with no signs of fluid overload along with a normal brain natriuretic peptide, this appeared to be less likely the cause. While waiting for an endobronchial ultrasound bronchoscopy (EBUS), the patient’s respiratory status continued to decline, requiring the cancellation of the EBUS. However, an osteolytic lesion on the T11 vertebrae was identified as a possible IR-guided biopsy site, and the patient was temporarily transferred to a tertiary hospital to receive the procedure on May 25, 2021 (Figure 3).

**Figure 4:**
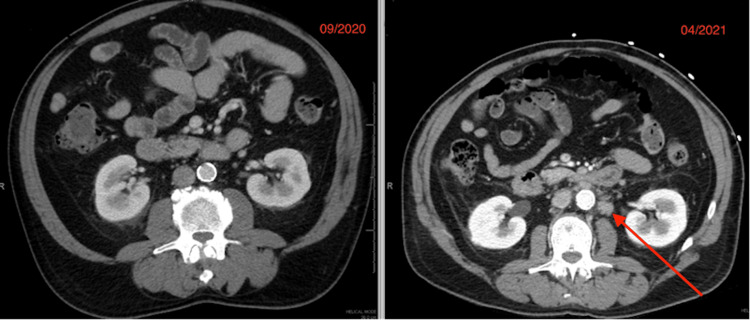
CT Abdomen and pelvis lymphadenopathy in comparison to prior imaging

While the biopsy results were pending, the patient’s clinical status continued to deteriorate. Repeat infectious workup was obtained, including blood and sputum cultures, a respiratory viral PCR panel, strep pneumonia, legionella urinary antigens, as well as mycoplasma pneumonia serum antigen; however, no infectious etiology was identified. A CT abdomen and pelvis were completed, which showed a significant increase in abdominal lymphadenopathy in comparison to seven months prior (Figure 4).

**Figure 5:**
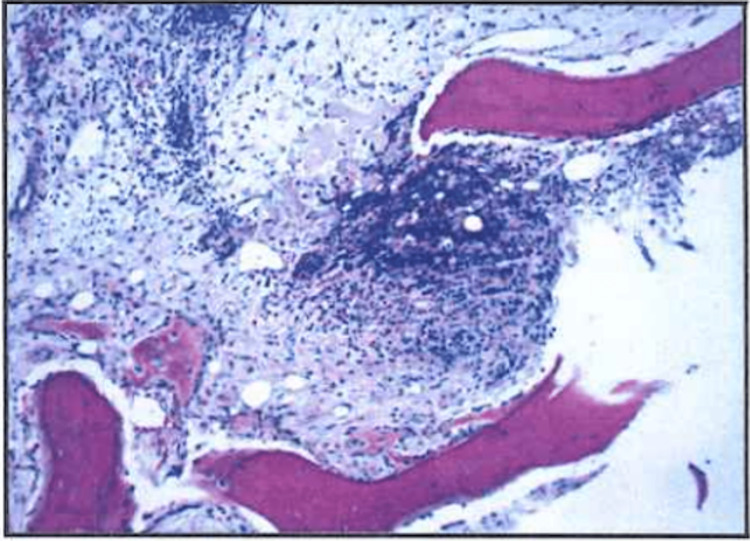
H&E (hematoxylin and eosin) stain showing poorly differentiated carcinoma with no glandular differentiation

However, the patient’s respiratory status was acutely declining, and the patient required self-proning and a non-rebreather to maintain adequate oxygenation. Biopsy results returned three days later, which showed metastatic, poorly differentiated carcinoma, possibly of urothelial origin. Microscopically the sections showed poorly differentiated carcinoma with nuclear hyperchromasia and an increase in the CD (cluster of differentiation) ratio with no obvious glandular differential (Figure 5).

**Figure 6:**
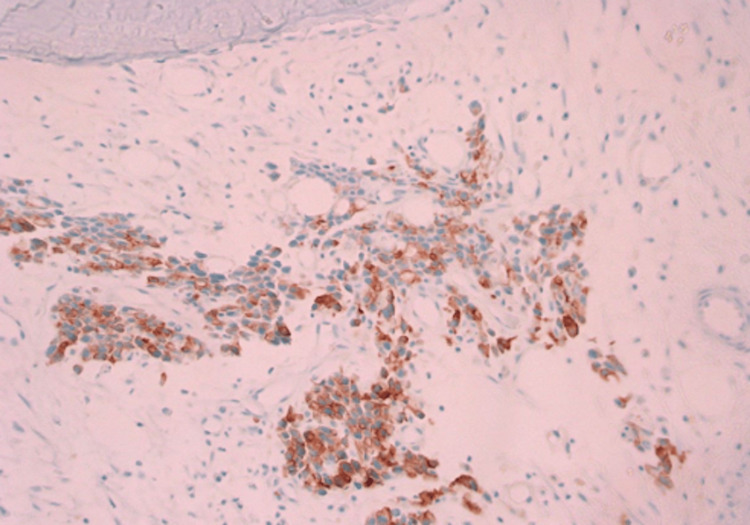
Positive CK7 stain

**Figure 7:**
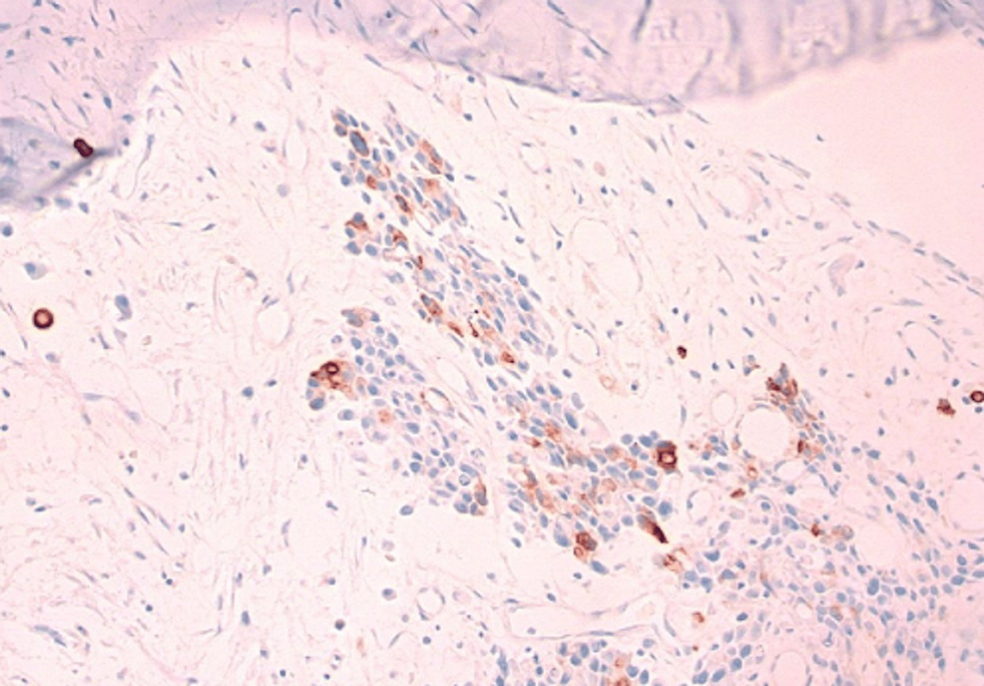
Positive CK20 stain

Immunohistochemical panel showing tumor cells positive for CK7 and CK20 but negative for PSA, CDX2, TTF-1, Napsin-A, GATA-3, chromogranin, and synaptophysin (Figures 6, 7).

Due to the delay in the availability of inpatient chemotherapy at our hospital, the decision was made to transfer the patient to a tertiary hospital to receive urgent chemotherapy due to his rapid respiratory decline without any identifiable source for hypoxia other than the malignancy. Unfortunately, over the next few hours, the patient was intubated for acute hypoxic respiratory failure and eventually went into cardiac arrest without successful resuscitation. The family was offered a biopsy to further investigate the cause of the patient’s death but respectfully declined.

## Discussion

Since the outbreak of the COVID-19 pandemic, there has been increasing literature regarding the lingering long-term effects of a COVID-19 infection. These symptoms may present with non-specific symptoms such as fatigue, breathlessness, cough, chest pain, palpitations, and headaches that persist months to years after initial inoculation [6]. Recent literature hypothesizes that a change in a body’s immune response may contribute to these symptoms but may also predispose patients to higher cancer rates or more rapid progression.

The progression of both solid and hematological malignancies is due to the complex interactions between cytokines, chemokines, and growth factors [7]. Though there is a vast amount of literature describing the relationship between these factors and oncogenesis, there remain limited studies regarding how inflammatory cytokines are modulated in cancer patients after SARS-CoV-2 infection/exposure. Several pro-inflammatory cytokines, such as IL-6 and TNF-a, have been shown to be upregulated in SARS-CoV-2 infection, which is theorized to alter the rate of cancer progression [8-10].

A study by Winter et al. [11] aimed to assess the relationship between the COVID inflammatory response in patients with an established cancer diagnosis. They found that at baseline, cancer patients had intrinsically high levels of inflammatory cytokines, which escalated significantly after a SARS-CoV-2 infection. In fact, patients with hematological malignancies showed a sustained dysregulated immune response persisting for up to three months [12]. 

Another study found that SARS-CoV-2 can cause activation of JAK-STAT, MAPK, and NF-kappaB, which have been known as part of the oncologic pathways that promote cancer cell development [13]. Other researchers have found that non-structural protein 3 (Nsp3), which is part of the COVID virus protein, has been reported to cause degradation of the tumor suppressor gene p53, while another protein, Nsp15, may interact with the retinoblastoma tumor suppressor gene (Rb) and subsequently cause down-regulation of Rb via the ubiquitin-proteasome pathway [14-15]. Recent evidence has also reported evidence of aberrant immune cell crosstalk in metastasis formation between inflammatory pathways [15].

Through similar mechanisms, our patient may have developed an exaggerated progression of his cancer due to the elevated cytokine response. After contracting COVID, he may have had an upregulatory response in several cytokines, resulting in a sustained inflammatory response even months later when he came back with shortness of breath. As a result of his dysregulated immune response, his respiratory decline likely happened more rapidly than to be expected compared to a cancer patient who had never contracted COVID. In fact, this hypothesis is also supported through the findings of another study, which analyzed leukocyte dysregulation through flow cytometry in cancer patients who were exposed to COVID-19 [16]. In their article, they noted in their results that COVID-19 positive cancer patients showed expression of exhaustion markers by CD8+ T cells. This phenomenon of exhausted T cells may compromise virus clearance, consistent with the observation that >70% of COVID-19 positive cancer patients displayed prolonged viral persistence. In fact, their findings suggested that through their observations, combined with the large registry studies, the principal cause of elevated mortality risk from COVID-19 in solid cancer patients is cancer progression. 

In conjunction with the high mortality associated with lymphangitic carcinomatosis, more case reports are needed to identify the strength of the correlation between the two entities. 

## Conclusions

Unfortunately, in the above case, due to the rapid decline of the patient’s respiratory status, further invasive workup was limited regarding his lung findings. However, what we can infer is that it may be possible that the patient already had a significant increase in inflammatory response after his first exposure to COVID, which continued to be sustained towards his second hospitalization. His persistent inflammatory response allowed for the progression of the carcinomatosis to occur much more rapidly than expected compared to a non-COVID-exposed patient.

This case highlights how our understanding of lymphangitic carcinomatosis post-COVID exposure is still very limited, and further case reports and research should be published to highlight this rare entity. Altogether, physicians and other medical professionals should be aware of the clinical presentations of lymphangitic carcinomatosis and expedite their diagnostic workup as timely as they can due to proposed worse outcomes in patients with a history of COVID infection.
